# Detection of differentially expressed genes in spatial transcriptomics data by spatial analysis of spatial transcriptomics: A novel method based on spatial statistics

**DOI:** 10.3389/fnins.2022.1086168

**Published:** 2022-11-29

**Authors:** Zhihua Qiu, Shaojun Li, Ming Luo, Shuanggen Zhu, Zhijian Wang, Yongjun Jiang

**Affiliations:** ^1^Nanfang Hospital, Southern Medical University, Guangzhou, Guangdong, China; ^2^Department of Neurology, The Second Affiliated Hospital of Guangzhou Medical University, Guangzhou, China; ^3^Department of Neurology, People’s Hospital of Longhua, Shenzhen, China

**Keywords:** spatial transcriptomics, DEGs, spatial statistics, saSpatial, stroke

## Abstract

**Background:**

Spatial transcriptomics (STs) simultaneously obtains the location and amount of gene expression within a tissue section. However, current methods like FindMarkers calculated the differentially expressed genes (DEGs) based on the classical statistics, which should abolish the spatial information.

**Materials and methods:**

A new method named spatial analysis of spatial transcriptomics (saSpatial) was developed for both the location and the amount of gene expression. Then saSpatial was applied to detect DEGs in both inter- and intra-cross sections. DEGs detected by saSpatial were compared with those detected by FindMarkers.

**Results:**

Spatial analysis of spatial transcriptomics was founded on the basis of spatial statistics. It was able to detect DEGs in different regions in the normal brain section. As for the brain with ischemic stroke, saSpatial revealed the DEGs for the ischemic core and penumbra. In addition, saSpatial characterized the genetic heterogeneity in the normal and ischemic cortex. Compared to FindMarkers, a larger number of valuable DEGs were found by saSpatial.

**Conclusion:**

Spatial analysis of spatial transcriptomics was able to effectively detect DEGs in STs data. It was a simple and valuable tool that could help potential researchers to find more valuable genes in the future research.

## Introduction

Spatial transcriptomic (ST) is a revolutionary technology that enables us to obtain information on spatial location and amount of gene expression within a tissue section (Stahl et al., 2016). It provides us with a unique opportunity to elucidate the cellular heterogeneity in tissues with a variety of biological conditions such as tumor and ischemia. Thus, enormous progress in the fields of embryology, oncology, and neuroscience has been made in recent years (Maniatis et al., 2019; Baccin et al., 2020).

Detection of differentially expressed genes (DEGs) among the different biological situations is the fundamental for microarray analysis including ST (Dries et al., 2021). Tools such as FindMarkers or FindAllMarkers, originally designed for single-cell RNA sequencing (scRNA-seq), have been applied in ST data (Stahl et al., 2016). FindMarkers is based on the Wilcoxon rank-sum test, a non-parametric test that belongs to classical statistics (Dutta and Datta, 2016). The essential assumption of classical statistics is that each individual must be independent (Griffith, 2005). Gene expression of one spot is very likely to be affected by the nearby ones, which indicates that the individuals of ST data are not independent. It has been demonstrated by recent studies (Svensson et al., 2018; Xia et al., 2019). In this way, the ST data was not suitable for classical statistics. It is necessary to develop a novel method for DEGs detection that simultaneously considers spatial context and gene expression.

Spatial statistics, or called spatial analysis, is a method primarily used in Geography to analyze the effect of spatial location on certain features (Griffith, 2005). For Health science, it is widely used in epidemiology. Unlike classical statistics, there is an essential assumption in spatial statistics: spatial data gathered from nearby areas are dependent on each other. Therefore, recent studies have noted the value of spatial statistics on the analysis of ST data (Xia et al., 2019; Hirz et al., 2022). There are a lot of parameters in spatial statistics and Moran’s index (Moran’s *I*) is a measure of spatial autocorrelation, which is characterized by a correlation in a signal among nearby locations. Moran’s *I* are classified into two categories: global and local. The global Moran’s *I* is a measure of the overall clustering of the spatial data, and the local Moran’s *I* is developed to evaluate the local spatial autocorrelation analysis (Li and Calder, 2007). Previous studies used the global Moran’s *I* to determine if there was global autocorrelation of ST data (Xia et al., 2019; Hirz et al., 2022). How to explore the DEGs *via* spatial statistics remained unclear.

Spatial statistics can be performed using geographical information systems (GIS) (Kistemann et al., 2002). Direct application of GIS for detection of DEGs in ST data was not yet available. Here we described a method named spatial analysis of spatial transcriptomics (saSpatial) to identify the DEGs in both inter- and intra-cross sections.

## Materials and methods

### Animal preparation

Adult C57BL/6 mice (8 weeks old, 25–35 g), provided by Animal Center of Southern Medical University (Guangzhou, China), were used in the present study. The animals were housed for at least 1 week before surgery under controlled environmental conditions with ambient temperature of 25°C, relative humidity of 65%, and 12/12 h light-dark cycle. The animals were free access to gain food and water. The protocol was reviewed and approved by the Institution Animal Care and Use Committee of The Second Affiliated Hospital of Guangzhou Medical University (NO. B2019-037).

### Distal MCAO mouse model

The mice were anesthetized using isoflurane (1–2%/oxygen). A cortical stroke model was made by occlusion of the distal middle cerebral artery using a previously reported method (Wen et al., 2019). In brief, a 1 cm skin incision was made between the ear and eye (usually right side). The temporal muscle was removed from the skull. A hole was made by the drill right above the MCA and the artery was coagulated by the electrocoagulation forceps proximal and distal to the bifurcation. The temporal muscle was relocated to cover the burr hole. Suture the wound and place the animal in a nursing box at 32°C to recover from anesthesia and return it to the cage.

### Magnetic resonance imaging

Magnetic resonance imaging (MRI) scanning was performed 6 h after stroke onset using a Bruker Biospec 7.0 T system (PharmaScan, Bruker Biospin, Rheinstetten, Germany) with a mouse brain array coil and a transmit only volume coil. The anesthetized animals were secured within the cradle by tooth and ear bars, and a mouse head four-channel phased array surface receiver coil was placed on the head. Body temperature was maintained at 37 ± 0.5°C during the MRI scanning procedure by a closed circuit thermal jacket.

T2 weighted scans using a fast-spin echo sequence: echo time (TE) 33 ms, repetition time (TR) 8,000 ms, field of view (FOV) 30 mm × 30 mm, acquisition matrix 512 × 512, acquiring 0.4 mm thick slices. A four-shot spin-echo planar imaging DWI scan (TE 30.5 ms, TR 8,000 ms, FOV 25 mm × 25 mm, acquisition matrix 96 × 96, three directions x, y, z, *B*-values = 0 and 1,000 s/mm^2^, 50 contiguous thick, 0.4 mm thick). The PWI can be performed non-invasively by tagging protons in the arterial blood supply with an inversion pulse by using a T2*-weighted echo-planar sequence: TE 76 ms, TR 11,482 ms, FOV 24 mm × 24 mm, acquisition matrix 128 × 128, 1 excitation, repetition 50 times. All MR images were processed on a commercial workstation (ParaVision Acquisition 6.0.1).

### Spatial transcriptomics

#### Tissue section preparation

The mice were sacrificed under deep anesthesia immediately after MRI scanning. The animals were perfused transcardially with 0.1 M PBS (pH 7.4) only, and brains were surgically removed rapidly and embedded in optimal cutting temperature (OCT) compound (SAKURA). The brains with OCT compound were quick-frozen on dry ice immediately and stored at −80°C until cryosectioning process. The cryosectioning placed in a cryostat (Leica, CM1950) to cryosection the OCT embedded tissue blocks into appropriately sized sections for Visium Spatial slides while keeping the samples frozen. Tissue sections were 10 μm thick each. Tissue sections were placed within the frames of Capture Areas on Visium Spatial slides (10X Genomics).

#### Fixation, staining, and imaging

Tissue section slides were incubated 1 min at 37°C then were fixed in methanol at −20°C for 30 min. For staining, the slides were incubated in Hematoxylin for 7 min and in Bluing Buffer for 2 min. Then, Eosin was added to the slides and incubated for 1 min. After each staining steps, slides were washed with DNase and RNase free water. Stained tissue sections are imaged by the microscope (Pannoramic MIDI, 3DHISTECH).

#### Tissue pre-permeabilization

Pre-permeabilization was performed to optimal the suitable permeabilization time. Visium Spatial Tissue Optimization Slides and Reagent Kits (10X Genomics) were used for pre-permeabilization. The tissues were permeabilized in Permeabilization Enzyme for varying amounts of time then the Fluorescent RT Master Mix was added to the tissue sections. For tissue removal, the tissue sections were incubated in Tissue Removal Mix for 60 min at 56°C. The best permeabilization time was selected through the fluorescent microscope (Pannoramic MIDI, 3DHISTECH).

#### Tissue permeabilization and spatial transcriptomic sequencing

Tissue permeabilization and ST sequencing were performed using Visium Spatial Gene Expression Slides and Reagent Kits. The stained slides were incubated in RT Master Mix for 45 min at 53°C for reserve transcription after permeabilization for appropriate time. Next, Second Strand Mix was added to the tissue sections on the slide and incubated for 15 min at 65°C to initiate second strand synthesis. After transfer of cDNA from the slides, barcoded-cDNA was purified and amplified. The amplified barcoded cDNA was fragmented, A-tailed, ligated with adaptors and index PCR amplified. The final libraries were quantified using the Qubit High Sensitivity DNA assay (Thermo Fisher Scientific, Waltham, MA, USA) and the size distribution of the libraries were determined using a High Sensitivity DNA chip on a Bioanalyzer 2200 (Agilent). All libraries were sequenced by illumina sequencer (Illumina, San Diego, CA, USA) on a 150 bp paired-end run.

#### Differentially expressed genes detected by spatial transcriptomic analysis

We applied fastp with default parameter filtering the adaptor sequence and removed the low-quality reads to achieve the clean data (Chen et al., 2018). Then the feature-barcode matrices were obtained by aligning reads to the mouse genome using SpaceRanger v1.1.0. In order to minimize the sample batch, we applied the down sample analysis among samples sequenced according to the mapped barcoded reads per spot of each sample and finally achieved the aggregated matrix.

Seurat package (version: 3.2)^1^ was used for spot normalization and regression. PCA was constructed based on the scaled data with all high variable genes. Utilizing graph-based cluster method, we acquired the unsupervised the cell cluster result based the PCA top 10 principal and we calculated the marker genes by FindAllMarkers function with Wilcoxon rank sum test algorithm under following criteria: logFC > 0.25; *p*-value < 0.05; min.pct > 0.1.

### Process of spatial analysis of spatial transcriptomics

#### Construction of spatial map

(a) Read a file with the.bz2 extension in the ST data folder including the contents of gene expression and coordinate of each individual spot (R Script 1).

(b) Export the data to a CSV file with columns of Barcode ID, gene name, standardized expression level (named as 1.csv), and another CSV file with x- and y-coordinate (named 2.csv) (R Script 1).

(c) Modify the coordinates in the 2.csv file. The value of x-coordinate was plus 40,531,000 and the value of y-coordinate is plus 3,460,000.

(d) Construct the spatial map using software Arcmap 10.8 (ESRI 2019. ArcGIS Desktop: Release 10.8. Redlands, CA, USA: Environmental Systems Research Institute). Create a new map document and choose the Add XY Data; import the 2.csv; the coordinate system was using CGCS2000_3_Degree_GK_Zone_40; export the spatial map data to a file with extension.shp (named without.shp in our experiment).

#### Data pre-processing

(a) Spilt the 1.csv into files, each of which include one single gene expression. The files are usually named as gene name like A_AQP4.xlsx following the gene of *Aqp4*, a marker of astrocyte cell. It should be added with “A_” before the gene name. It is realized by R Script 2.

(b) Each excel filename is read by Python Script 1 to exclude the potential errors.

(c) Create a new document in Arcmap 10.8 and import all the excel files by Python Script 2. The document is named and saved as T.gdb.

(d) Create another new map document named Joined.gdb. Add T.gdb to without.shp. This adds the gene expression information to spatial map. It was realized by Python Script 3.

#### Global Moran’s index calculation

(a) The global Moran’s *I* statistic is given as:


$$I=\frac{n}{S_{0}}\,\frac{\sum_{i=1}^{n}\sum_{j=1}^{n}\omega_{i,j}\,Z_{i}\,Z_{j}}{\sum_{i=1}^{n}Z_{i}^{2}}$$

where *Z*_*i*_ is the deviation of an attribute for feature I from its mean (*X*_*i*_-$\bar{X}$), ω_*i,j*_ is the spatial weight between feature *i* and *j*, n is equal to the total number of features, and *S*_*0*_ is the aggregate of all the spatial weights:


$$S_{0}=\sum_{i=1}^{n}\sum_{j=1}^{n}\omega_{i,j}$$

The *Z*_*i*_-score for the statistic is computed as:


$$Z_{i}=\frac{I-E\,\left[I\right]}{\sqrt{\nu \,\left[I\right]}}$$

where:


$$E\,\left[I\right]=\frac{-1}{n-1}$$

$$\nu \,\left[I\right]=E\,\left[I^{2}\right]-E\,{\left[I\right]}^{2}$$

It was realized by Python Script 3.

(b) Extract the data of global Moran’s *I*, expected index, variance, z-score and *P*-value of each gene into one excel file (Python Script 4).

#### Local Moran’s *I* calculation

(a) The local Moran’s *I* statistic is given as:


$$I_{i}=\frac{X_{i}-\bar{X}}{S_{i}^{2}}\,\sum_{j=1,j\neq i}^{n}\omega_{i,j}\,\left(x_{j}-\bar{X}\right)$$

Where *X*_*i*_ is an attribute for feature i, $\bar{X}$ is the mean of the corresponding attribute, ω_*i*,*j*_ is the spatial weight between feature i and j, and:


$$S_{i}=\frac{\sum_{j=1,j\neq i}^{n}\omega_{i,j}\,{\left(x_{j}-\bar{X}\right)}^{2}}{n-1}$$

With n equating to the total number of features.

The *Z*_*I_i_*_-score for the statistics are given as:


$$Z_{I_{i}}=\frac{I_{i}-E\,\left[I_{i}\right]}{\sqrt{\nu \,\left[I_{i}\right]}}$$

where:


$$E\,\left[I_{i}\right]=-\frac{\sum_{j=1,j\neq i}^{n}\omega_{i,j}}{n-1}$$

$$\nu \,\left[I_{i}\right]=E\,\left[I_{i}^{2}\right]-E\,{\left[I_{i}\right]}^{2}$$

(b) The local Moran’s *I* of each spot is realized by Python Script 5 using the files of Step “Construction of spatial map” (d), Step “Data pre-processing” (c and d) (without.shp, T.gdb and Joined.gdb).

(c) The information includes local Moran’s *I*, *P*-value, *Z*_*I_i_*_-score and spatial cluster type (CO type).

#### Comparison between region of interest and other regions

(a) Using the spatial map constructed in the Step “Construction of spatial map,” the ROI is circulated. The spot barcode in the ROI is obtained.

(b) The number of high-to-high (H-H) or low-to-low (L-L) spots in the ROI is extracted and compare with other regions using Chi-Squared Test. It is realized by R Script 3.

(c) Top 20 genes of each comparison are used for bubble plot.

#### Visualization

(a) For any specific gene identified by the above analysis, add the CSV file to spatial map.

(b) Run Hot Spot analysis by Arcmap 10.8.

(c) Use the Legend, and convert it into graphics.

(d) Export the figure.

## Results

### Flow of spatial analysis of spatial transcriptomics method

**FIGURE 1:**
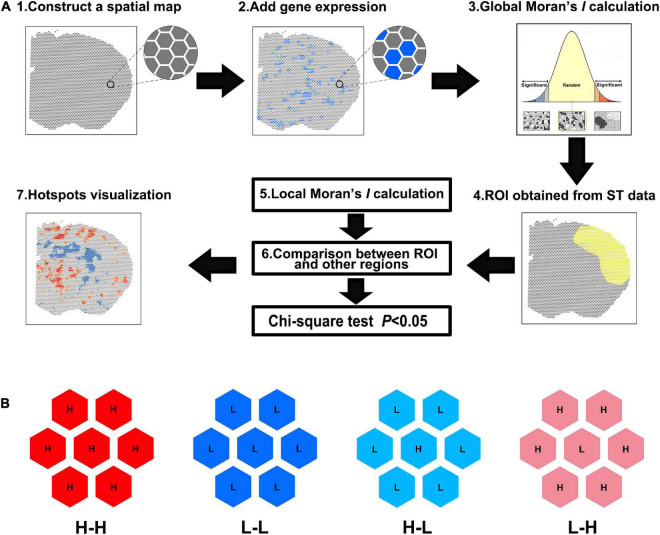
Spatial analysis of spatial transcriptomics (saSpatial) flow chart. **(A)** Flow chart of saSpatial. **(B)** Spatial cluster type. ST, spatial transcriptomics; ROI, region of interest; H-H, high-to-high; L-L, low-to-low; H-L, high-to-low; L-H, low-to-high.

As shown in Figure 1A, the spatial maps were constructed based on the spot barcodes derived from the ST data. The gene expression quantities were then connected to the corresponding spots. Global Moran’s *I* was calculated to determine whether it was clustered. If not, the gene was aborted for further analysis. Next, the ROI was circled in the ST maps and the spot barcodes of the ROI were obtained. Then, local Moran’s *I* was used to determine the CO type, which was group into five categories: non-significant, H-H, L-L, high-to-low (H-L), and low-to-high (L-H) spots (Figure 1B). The number of H-H and/or L-L spots were used for the following statistics. Finally, visualization of DEGs was performed using Hot Spot analysis with Arcmap 10.8.

### Spatial autocorrelation patterns revealed by global Moran’s *I*

**FIGURE 2:**
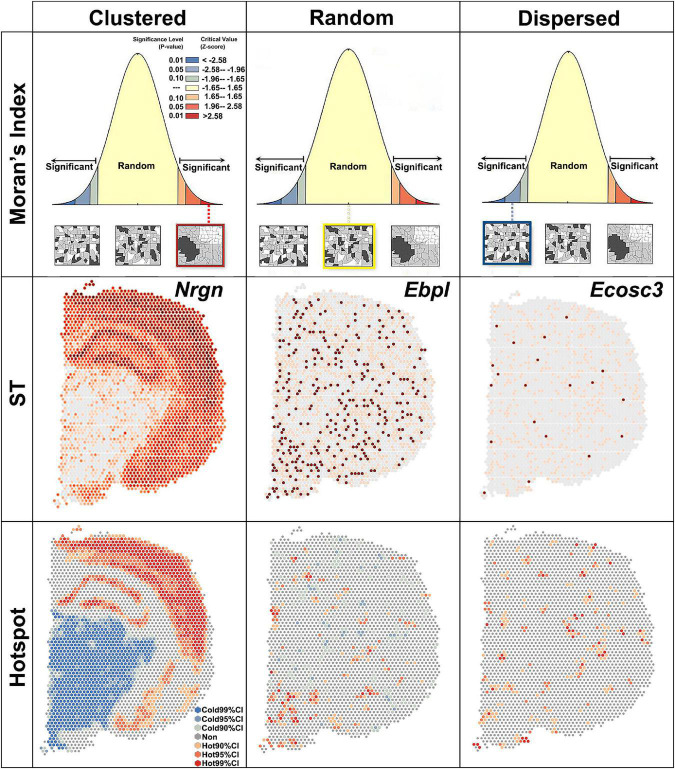
Global Moran’s index revealing three spatial autocorrelation patterns. Clustered, *z* > 1.65 and *P* < 0.1; random, –1.65 < z < 1.65 and *P* > 0.1; dispersed, z < –1.65 and *P* < 0.1. ST, spatial transcriptomic; CI, confidence interval.

Global Moran’s *I* measures the spatial autocorrelation based on both the locations and the quantities of features. A value of 0 for Moran’s *I* indicated no autocorrelation. In the brain section, no gene showed no autocorrelation. Z-score and *P*-value were used to evaluate the significance of global Moran’s *I* (Figure 2). Based on the global Moran’s *I*, z-score and *P*-value, the spatial pattern were group into: clustered, random and dispersed (Figure 2). If global Moran’s *I* was close to + 1 and z-score was more than 1.65, the pattern was clustered, which means elevated gene expression had similar elevation values close to each other like *Nrgn* (Figure 2). If global Moran’s I was close to −1 and z-score was less than −1.65, the pattern was dispersed, which means dissimilar values were next to each other like a checkerboard (Figure 2).

### Differentially expressed genes of different regions in normal brain revealed by spatial analysis of spatial transcriptomics

**FIGURE 3:**
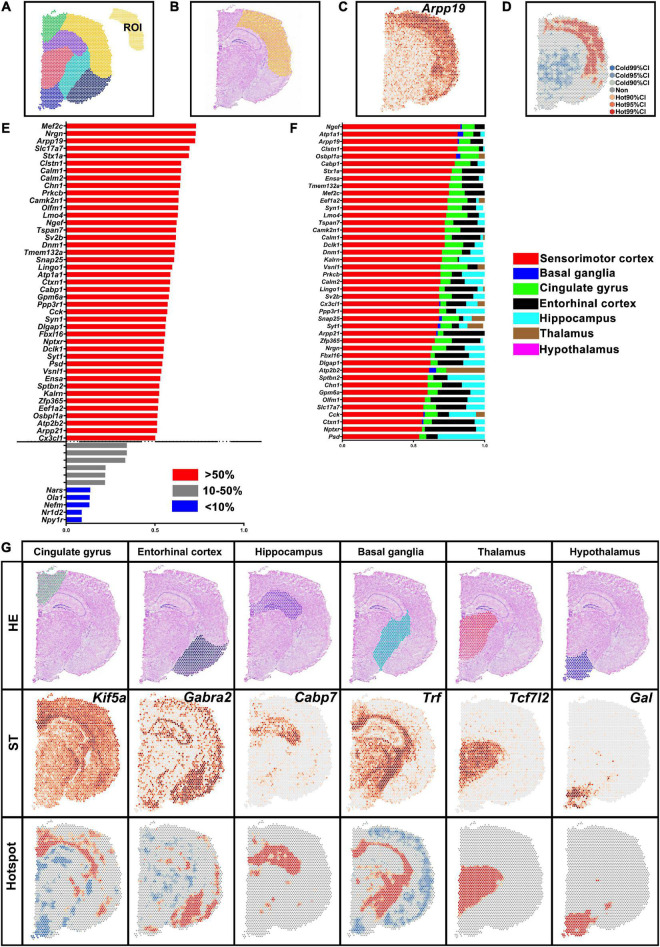
Differentially expressed genes (DEGs) in the different brain regions. **(A)** Brain region. ROI indicates sensorimotor cortex. **(B)** Yellow area indicates sensorimotor cortex in HE images. **(C)** *Arpp19* of ST map. **(D)** Hotspot of *Arpp19*. **(E)** DEGs in the sensorimotor cortex. Ratio = The number of H-H spots in sensorimotor cortex/The total number of spots in the section. **(F)** Septicity of DEGs in the sensorimotor cortex. Ratio = The number of H-H spots in each region/The total number of H-H spots. **(G)** Marker genes of each brain region. ST, spatial transcriptomic.

Spatial transcriptomic data of normal brain section was obtained from the 10X Genomics dataset. The brain hemisphere was divided into sensorimotor cortex, basal ganglia, cingulate gyrus, entorhinal cortex, hippocampus, thalamus, and hypothalamus (Figure 3A). The ratio of H-H spots in the sensorimotor cortex was calculated and is shown in Figure 3E. Then we determined the proportion of H-H spots (more than 50% in Figure 3E) in the other brain regions (Figure 3F). For instance, *Arpp19* mostly enriched in the sensorimotor cortex while it also was found in the cingulate gyrus and entorhinal cortex, which was verified in ST map (Figures 3B–D,F). DEGs in other brain regions were also identified and typical genes are shown in Figure 3G. All these results indicated that saSpatial was able to detect DEGs of ROI within a section.

### Up-regulated differentially expressed genes of ischemic penumbra and core

**FIGURE 4:**
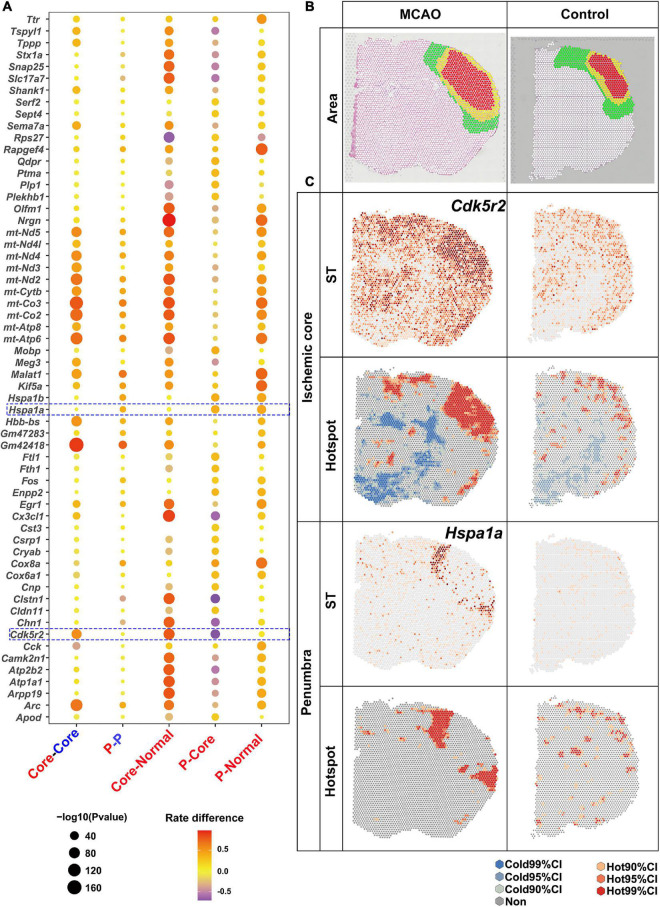
Upregulated DEGs revealed by saSpatial. **(A)** Bubble plots of top20 DEGs. *P*-values are indicated by circle size; scale adjacent to the plot. The rate difference between stroke and NC group is indicated by color. NC, normal control. P, penumbra. **(B)** Ischemic brain area. Ischemic core (red), penumbra (yellow), and normal area (green) in the sensorimotor cortex. **(C)** Typical DEGs of ischemic core and penumbra. *N* = 3. CI, confidence interval.

Spatial analysis of spatial transcriptomics was then applied to determine the DEGs in the brain section from ischemic stroke. Ischemic stroke was induced in a mouse model by occlusion of the distal middle cerebral artery. MRI scanning was performed 6 h after stroke onset to determine the ischemic penumbra (mismatch of perfusion- and diffusion-weighted imaging) and core. Penumbra has been defined as brain tissue at a risk of infarction (Albers et al., 2018). The brains were then harvested for ST sequencing. Ischemic core and penumbra and the corresponding area in the normal brain section were marked with reference to HE and MRI images (Figure 4B).

Considering numerous gene expressions were similar in the ischemic penumbra and core, which was also proved by the following data (Figure 4A), there were three comparisons of the number of H-H spots: between the ischemic penumbra or core and the corresponding area in the normal brain sections; between the ischemic penumbra or core and the normal region in the ischemic stroke brain section; and between ischemic core and penumbra (Figure 4A). Bubble plots of the top 20 DEGs for each comparison identified by saSpatial are shown in Figure 4A. For instance, *Gm42418* was highly expressed in the ischemic hemisphere while there was no significant difference between ischemic ore and penumbra. In contrast, *Cdk5r2* was significantly increased in the ischemic core but not in the penumbra (Figure 4C). Hence, *Cdk5r2* but not *Gm42418* was a marker gene for ischemic core. As for ischemic penumbra, saSpatial identified the up-regulated DEGs like *Hspa1a*, which was like a thin loop around the core (Figure 4C).

### Down-regulated differentially expressed genes of ischemic penumbra and core

**FIGURE 5:**
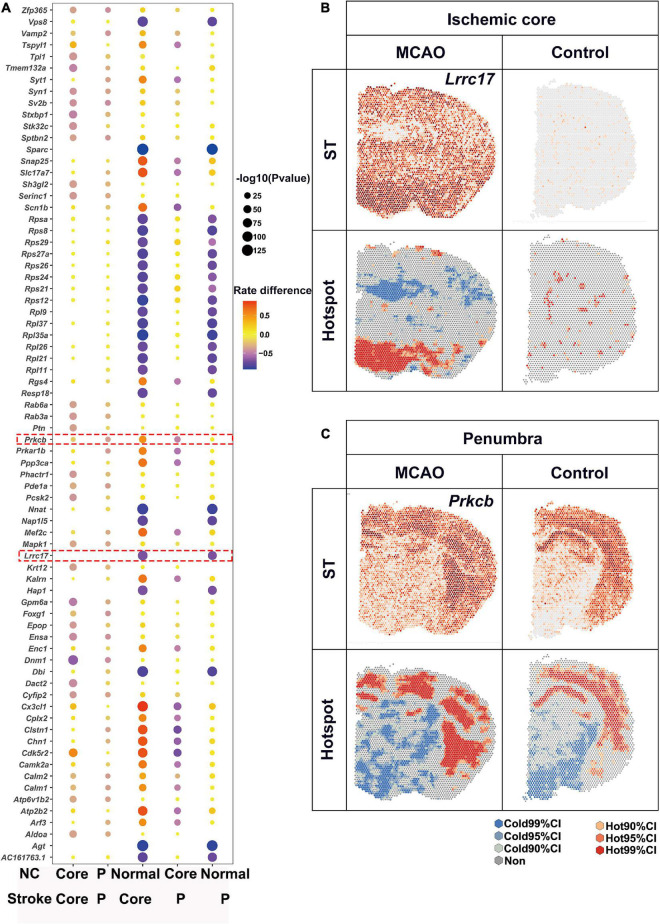
Downregulated DEGs revealed by saSpatial. **(A)** Bubble plots of top20 DEGs. *P*-values are indicated by circle size; scale adjacent to the plot. The rate difference are indicated by color. NC, normal control. P, penumbra. Typical DEGs of **(B)** ischemic core and **(C)** penumbra. *N* = 3. ST, spatial transcriptomic; CI, confidence interval.

Substantial attention was paid to the up-regulated expression, while expression of numerous genes were halted by ischemia. In contrast to the up-regulated DEGs as described above, the number of L-L spots was used for detection of down-regulated DEGs. Bubble plots of the top 20 DEGs for each comparison are shown in Figure 5A. There were a lot of genes down-regulated in ischemic area while most of them showed no significant differences between ischemic core and penumbra such as *Vps8* and *Sparc* (Figure 4A). For the ischemic core, *Lrrc77* was a marker gene (Figure 5B). As for the ischemic penumbra, down-regulated DEGs like *Prkcb* were identified (Figure 5C).

### Comparation of spatial analysis of spatial transcriptomics with FindMarkers

**FIGURE 6:**
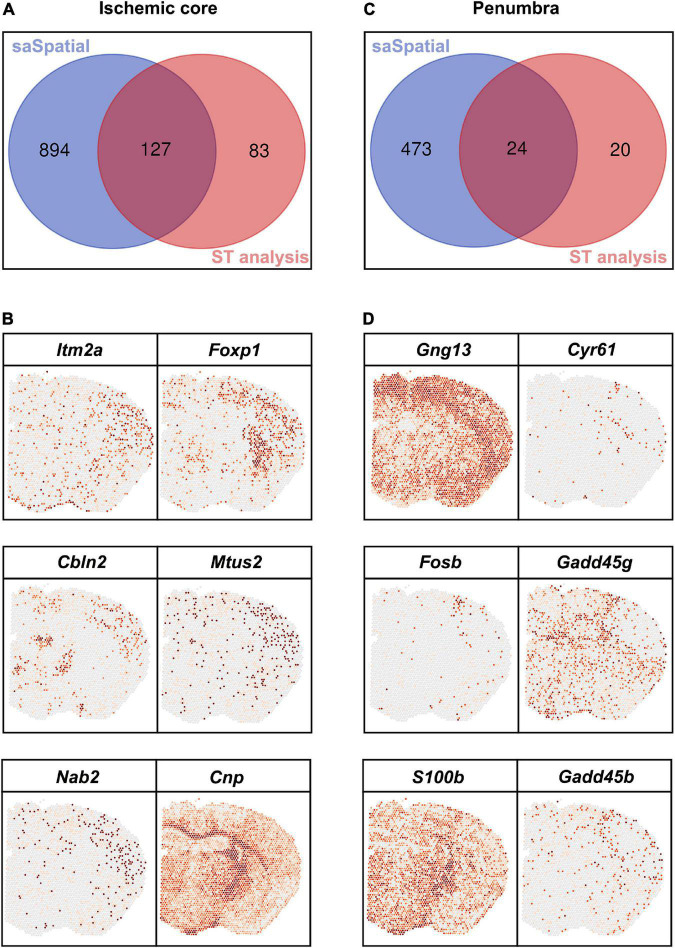
Comparison between saSpatial and FindMarkers. **(A)** Venn map of ischemic core. **(B)** DEGs only found by saSpatial in the ischemic core. **(C)** Venn map of penumbra. **(D)** DEGs only found by saSpatial in the penumbra. *N* = 3. CI, confidence interval.

Differentially expressed genes detected by saSpatial were compared with that detected by FindMarkers (Figure 6). For the ischemic core, FindMarkers found only 210 DEGs while there were 1,021 DEGs identified by saSpatial (Figure 6A and Supplementary Table 1). The majority of DEGs detected by FindMarkers could be also found by saSpatial (Figure 6A) while numerous genes like *Itm2a*, *Foxp1*, *Cbln2*, *Mtus2*, *Nab2*, and *Cnp* were not detected by FindMarkers (Figure 6B). For the ischemic penumbra, FindMarkers can only find 44 DEGs and the majority of DEGs were missed (Figure 6C). saSpatial found that there were 497 potentially valuable DEGs in the ischemic penumbra (Figure 6C) such as *Gng13*, *Cyr61*, *Fosb*, *Gadd45g*, *S100b*, and *Gadd45b*, which were confirmed by ST maps (Figure 6D).

### Differentially expressed genes of different cortical layers

**FIGURE 7:**
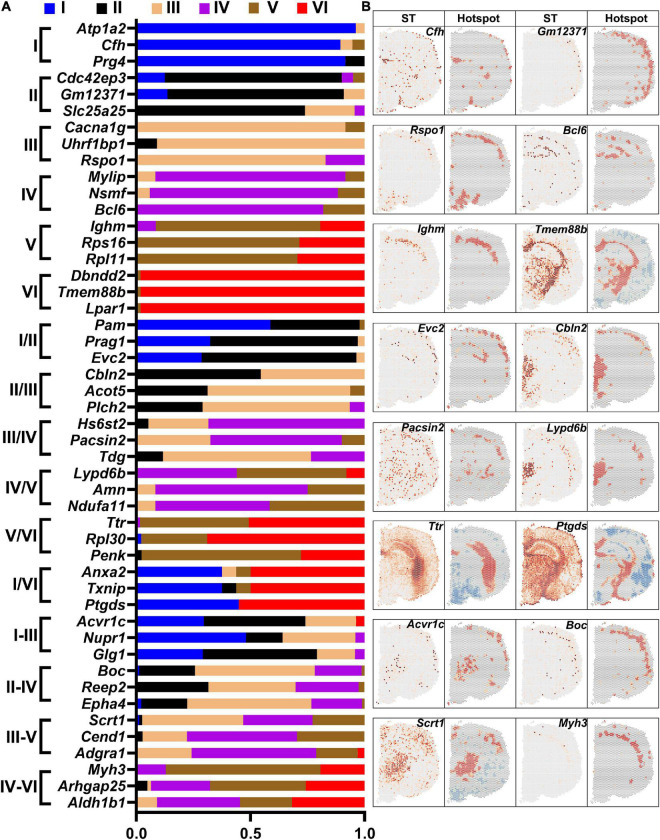
Differentially expressed genes (DEGs) in the cortex layers. **(A)** Top 3 DEGs in the layers. **(B)** Typical DEGs. ST, spatial transcriptomic.

The sensorimotor cortex is composed of six layers. DEGs in each layer were revealed by saSpatial and the top 3 DEGs in each layer are shown in Figure 7A. Layer V is the internal pyramidal layer and contains large pyramidal neurons, the axons of which form the corticospinal tract. saSpatial showed that *Ighm* was a marker gene in Layer V (Figure 7B).

### Differentially expressed genes of different cortical layers in the ischemic core

**FIGURE 8:**
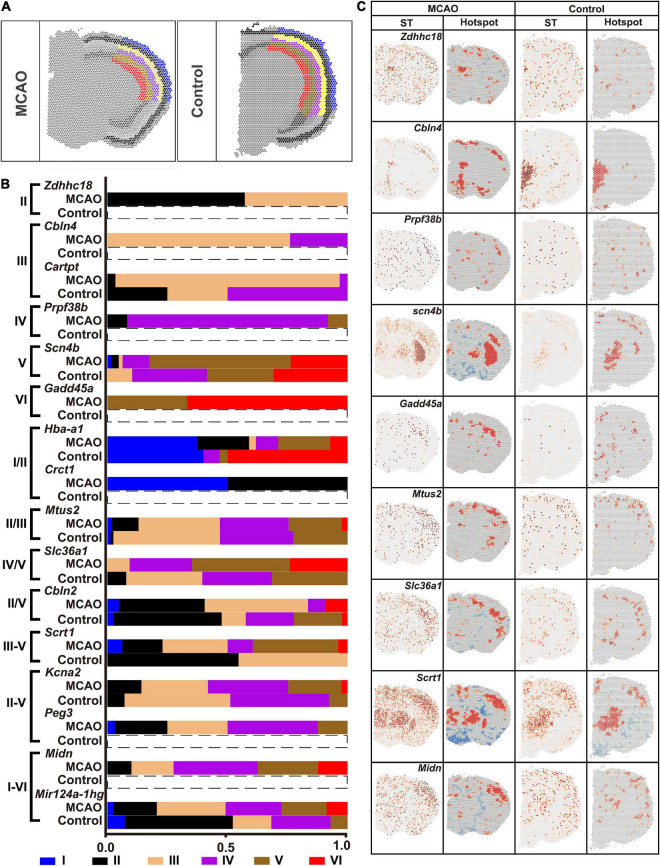
Heterogenicity of DEGs within the ischemic cortex. **(A)** Six layers of cortex in the ischemic cortex. **(B)** Typical DEGs. **(C)** ST map and Hotspot of typical DEGs. ST, spatial transcriptomic.

Different cortical layers responded differently to ischemic insult. Six layers of ischemic core and that in the normal brain section were marked with reference to the HE images (Figure 8A). The fraction of DEGs in each layer is shown in Figure 8B. The ST and Hotspot maps are shown in Figure 8C. For example, *Cbln4*, which was not found in the normal brain, was up-regulated in the ischemic core and enriched mainly in the Layer III. *Scn4b* was up-regulated in the Layer V while it was down-regulated in other layers (Figures 8B,C).

## Discussion

In the present study, we developed a simple, reliable and reproduceable method, named saSpatial, to detect the DEGs in ST data. DEGs from different biological situations within the same section or across the different sections can be clearly detected by saSpatial. Heterogenicity of a particular region can also be characterized by saSpatial. In comparison to the traditional method, saSpatial was able to detect more valuable DEGs.

Classical statistics-based methods such as FindMarkers detected the DEGs only referred to the value of gene expression (Dries et al., 2021). It neglects a basic fact that gene expression of cells is effected by the other cells especially the nearby ones. Several other methods such as SpatialDE have been developed to explore the variable genes from the spatial context while they failed to detect the DEGs of any specific ROI (Svensson et al., 2018; Xu and McCord, 2021; Zhu et al., 2021). Based on the spatial statistics, the CO types of any individual spot included information about both the effect of spatial location on gene expression and gene expression quantity. The chi-squared test was used to compare the number of H-H or L-L spots between the ROI and the other regions. This simple approach provided a tool for analyzing the ST data to detect the DEGs and characterize their heterogeneity within the ROI. To the best of our knowledge, it was the first tool to detect the DEGs in ST data with regard to location and value of gene expression simultaneously. We then used this tool to investigate the expression and distribution of genes in the normal and ischemic stroke brain sections.

Firstly, we used saSpatial to detect the DEGs in different regions of the normal brain section. Many previous studies have used ST to characterize the genetic heterogeneity in brain section. Usually, they used snRNA data to find the gene expressions in certain regions and projected the findings onto ST data like the multimodal intersection analysis (MIA) method (Baccin et al., 2020; Moncada et al., 2020). In contrast, saSpatial detected DEGs directly and quickly. In the present study, saSpatial found various DEGs in the different brain regions, such as *Arpp19* for sensorimotor cortex and *Cabp7* for hippocampus.

Then, saSpatial was used to detect the DEGs in the ischemic brain. Ischemic stroke was one of the leading causes of mortality and morbidity (Hankey, 2014). Ischemic insult resulted in significant changes of gene expressions (Xu et al., 2017). In the present study, saSpatial found various DEGs in the ischemic brain area. Ischemic brain area was divided into ischemic core and penumbra, of which DEGs was hard to distinguish. saSpatial can successfully distinguish the DEGs in the ischemic core and penumbra including the up- and down-regulated ones. Furthermore, more DEGs were found by saSpatial than that by FindMarkers.

Finally, saSpatial characterized heterogenicity of the gene expressions in the normal and ischemic cortex. Cortex genetic heterogenicity has been proved by many other studies (Stahl et al., 2016). Cortex was made up of six layers, each of which was a limited area like Layer I. How to identify the heterogenicity within a small region in ST data remained challenge. We used saSpatial and found that *Cfh* dominated in the Layer I and *Ighm* was the marker gene of Layer V. As for the ischemic cortex, *Cbln4* was up-regulated in the ischemic core and enriched mainly in the Layer III while *Scn4b* was up-regulated in the Layer V and down-regulated in other layers.

### Limitations

Spatial analysis of spatial transcriptomics was based on the ST data. Hence, the resolution of saSpatial relied on the raw ST data. A spot in ST data represented genes of an area including about 10 cells. Thus, saSpatial cannot reveal the cellular types. Secondly, CO types of spots mainly consider the effect of adjacent spots while some genes were affected by remote ones. Finally, the DEGs identified by saSpatial needs to be verified by the additional biological experiment.

## Conclusion

In conclusion, saSpatial was constructed based on local Moran’s *I* and successfully detected the DEGs in ST data. It would help the potential researchers to find more valuable DEGs in the future study.

## Data availability statement

The datasets used and/or analyzed during the current study are available from the corresponding author on reasonable request. ST data was available in the GEO site: https://www.ncbi.nlm.nih.gov/geo/query/acc.cgi?acc=GSE199066 and https://www.ncbi.nlm.nih.gov/geo/query/acc.cgi?acc=GSE199067. The code was provided in the Supplementary material.

## Author contributions

ZQ and SL collected the data and prepared the first draft. ML helped perform the animal model and MRI scanning. SZ and ZW analyzed as part of the data. YJ designed the whole study and approved the final manuscript. All authors contributed to the article and approved the submitted version.

## Footnotes

1
https://satijalab.org/seurat/

## References

[B1] Albers G. W. Marks M. P. Kemp S. Christensen S. Tsai J. P. Ortega-Gutierrez S. (2018). Thrombectomy for Stroke at 6 to 16 Hours with Selection by Perfusion Imaging. *N. Engl. J. Med.* 378 708–718. 29364767 10.1056/NEJMoa1713973 PMC6590673

[B2] Baccin C. Al-Sabah J. Velten L. Helbling P. M. Grunschlager F. Hernandez-Malmierca P. (2020). Combined single-cell and spatial transcriptomics reveal the molecular, cellular and spatial bone marrow niche organization. *Nat. Cell Biol.* 22 38–48. 10.1038/s41556-019-0439-6 31871321 PMC7610809

[B3] Chen S. Zhou Y. Chen Y. Gu J. (2018). fastp: An ultra-fast all-in-one FASTQ preprocessor. *Bioinformatics* 34 i884–i890. 10.1093/bioinformatics/bty560 30423086 PMC6129281

[B4] Dries R. Chen J. Del Rossi N. Khan M. M. Sistig A. Yuan G. C. (2021). Advances in spatial transcriptomic data analysis. *Genome Res.* 31 1706–1718. 34599004 10.1101/gr.275224.121 PMC8494229

[B5] Dutta S. Datta S. (2016). A rank-sum test for clustered data when the number of subjects in a group within a cluster is informative. *Biometrics* 72 432–440. 10.1111/biom.12447 26575695 PMC4870168

[B6] Griffith D. A. (2005). “Spatial Autocorrelation,” in *Encyclopedia of Social Measurement*, ed Kempf-Leonard K. (Amsterdam: Elsevier Science), 581–590

[B7] Hankey G. J. (2014). Secondary stroke prevention. *Lancet Neurol.* 13 178–194. 24361114 10.1016/S1474-4422(13)70255-2

[B8] Hirz T. Mei S. Sarkar H. Kfoury Y. Wu S. Verhoeven B. M. (2022). Integrated single-cell and spatial transcriptomic analyses unravel the heterogeneity of the prostate tumor microenvironment. *BioRxiv* [Preprint]. 10.1101/2022.03.18.484781 PMC9905093 36750562

[B9] Kistemann T. Dangendorf F. Schweikart J. (2002). New perspectives on the use of Geographical Information Systems (GIS) in environmental health sciences. *Int. J. Hyg. Environ. Health* 205 169–181. 12040915 10.1078/1438-4639-00145

[B10] Li H. C. A. Calder N. C. (2007). Beyond Moran’s I: Testing for Spatial Dependence Based on the Spatial Autoregressive Model. *Geogr. Anal.* 39 357–375.

[B11] Maniatis S. Aijo T. Vickovic S. Braine C. Kang K. Mollbrink A. (2019). Spatiotemporal dynamics of molecular pathology in amyotrophic lateral sclerosis. *Science* 364 89–93. 30948552 10.1126/science.aav9776

[B12] Moncada R. Barkley D. Wagner F. Chiodin M. Devlin J. C. Baron M. (2020). Integrating microarray-based spatial transcriptomics and single-cell RNA-seq reveals tissue architecture in pancreatic ductal adenocarcinomas. *Nat. Biotechnol.* 38 333–342. 31932730 10.1038/s41587-019-0392-8

[B13] Stahl P. L. Salmen F. Vickovic S. Lundmark A. Navarro J. F. Magnusson J. (2016). Visualization and analysis of gene expression in tissue sections by spatial transcriptomics. *Science* 353 78–82. 27365449 10.1126/science.aaf2403

[B14] Svensson V. Teichmann S. A. Stegle O. (2018). SpatialDE: Identification of spatially variable genes. *Nat. Methods* 15 343–346. 10.1038/nmeth.4636 29553579 PMC6350895

[B15] Wen Z. Jiang Y. Zhang L. Xu X. Zhao N. Xu X. (2019). The effect of anterior communicating artery flow on neurovascular injury and neurobehavioral outcomes in mice with recurrent stroke. *Brain Res.* 1724:146440. 10.1016/j.brainres.2019.146440 31513789

[B16] Xia C. Fan J. Emanuel G. Hao J. Zhuang X. (2019). Spatial transcriptome profiling by MERFISH reveals subcellular RNA compartmentalization and cell cycle-dependent gene expression. *Proc. Natl. Acad. Sci. U S A.* 116 19490–19499. 10.1073/pnas.1912459116 31501331 PMC6765259

[B17] Xu X. Wen Z. Zhao N. Xu X. Wang F. Gao J. (2017). MicroRNA-1906, a Novel Regulator of Toll-Like Receptor 4, Ameliorates Ischemic Injury after Experimental Stroke in Mice. *J. Neurosci.* 37 10498–10515. 10.1523/JNEUROSCI.1139-17.2017 28924010 PMC6596627

[B18] Xu Y. McCord R. P. (2021). CoSTA: Unsupervised convolutional neural network learning for spatial transcriptomics analysis. *BMC Bioinformatics* 22:397. 10.1186/s12859-021-04314-1 34372758 PMC8351440

[B19] Zhu J. Sun S. Zhou X. (2021). SPARK-X: Non-parametric modeling enables scalable and robust detection of spatial expression patterns for large spatial transcriptomic studies. *Genome Biol.* 22 184. 10.1186/s13059-021-02404-0 34154649 PMC8218388

