# Correction: Detection of differentially expressed genes in spatial transcriptomics data by spatial analysis of spatial transcriptomics: A novel method based on spatial statistics

**DOI:** 10.3389/fnins.2026.1867303

**Published:** 2026-05-22

**Authors:** Zhihua Qiu, Shaojun Li, Ming Luo, Shuanggen Zhu, Yongjun Jiang, Zhijian Wang

**Affiliations:** 1 Nanfang Hospital, Southern Medical University, Guangzhou, Guangdong, China; 2 Department of Neurology, The Second Affiliated Hospital of Guangzhou Medical University, Guangzhou, China; 3 Department of Neurology, People's Hospital of Longhua, Shenzhen, China

**Keywords:** spatial transcriptomics, DEGs, spatial statistics, saSpatial, stroke

Author Zhijian Wang was erroneously not assigned as a corresponding author. The correct corresponding authors are Zhijian Wang and Yongjun Jiang.

The order of authors in the author list of the published paper was erroneously given as: Zhihua Qiu, Shaojun Li, Ming Luo, Shuanggen Zhu, Zhijian Wang and Yongjun Jiang.

The correct author list reads: Zhihua Qiu, Shaojun Li, Ming Luo, Shuanggen Zhu, Yongjun Jiang and Zhijian Wang.

The original version of this article has been updated.

